# Supplementary material for: Relational Integration Demands Are Tracked by Temporally Delayed Neural Representations in Alpha and Beta Rhythms Within Higher‐Order Cortical Networks
Source: Hum Brain Mapp. 2025 Jul 7;46(10):e70272. doi: 10.1002/hbm.70272 (PMC12231057; doi:10.1002/hbm.70272)
Supplement: Supplementary file 1 — Data S1. hbm70272‐sup‐0001‐Supinfo. [file HBM-46-e70272-s001.docx]

**Supplementary**

**
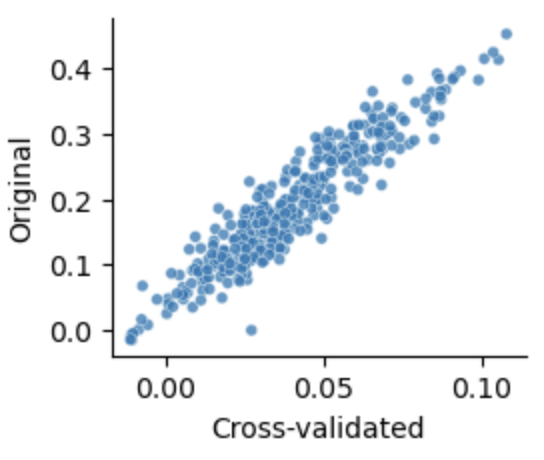
**

**Supplementary Figure 1.** *Correspondence between the average cross-validated (x-axis) and original (y-axis) average model-fit r-values for each brain region.*

**
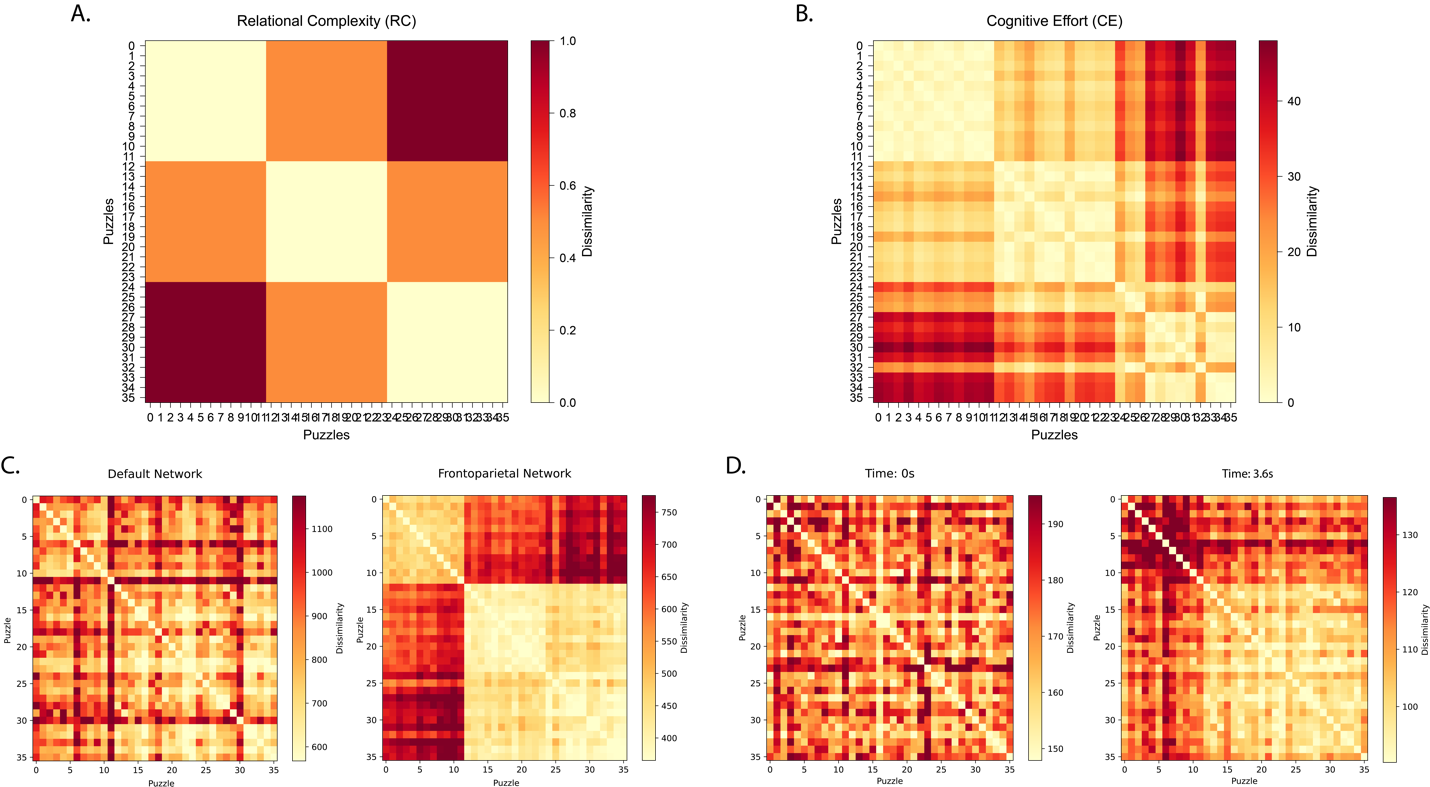
**

**Supplementary Figure 2**: *Model and empirical representational dissimilarity matrices (RDMs).* **A.** The relational complexity (RC) model defines puzzle dissimilarity based on the number of relations that must be integrated to reach a solution, with binary, ternary, and quaternary puzzles assigned values of 0.0, 0.5, and 1.0, respectively. **B.** The cognitive effort (CE) model is a data-driven model, derived by calculating the Euclidean distance between puzzles in a 2D feature space of error rate (1 – accuracy) and response time. Simplified cartoon illustrations are depicted in **Figures 1B** and **4A**. Both the RC and CE model RDMs were highly correlated (rs = 0.81, p<0.001). **C.** Empirical fMRI-based RDMs for the default mode (left) and frontoparietal (right) networks. **D.** Empirical EEG beta-band RDMs at puzzle onset (0 seconds; left) and at peak correspondence with the RC model (3.2 seconds; right). Each matrix depicts the pairwise dissimilarity between individual puzzles, where each row and column represents a single puzzle. Cooler colours (yellow) indicate higher similarity, and warmer colours (dark red) indicate greater dissimilarity.


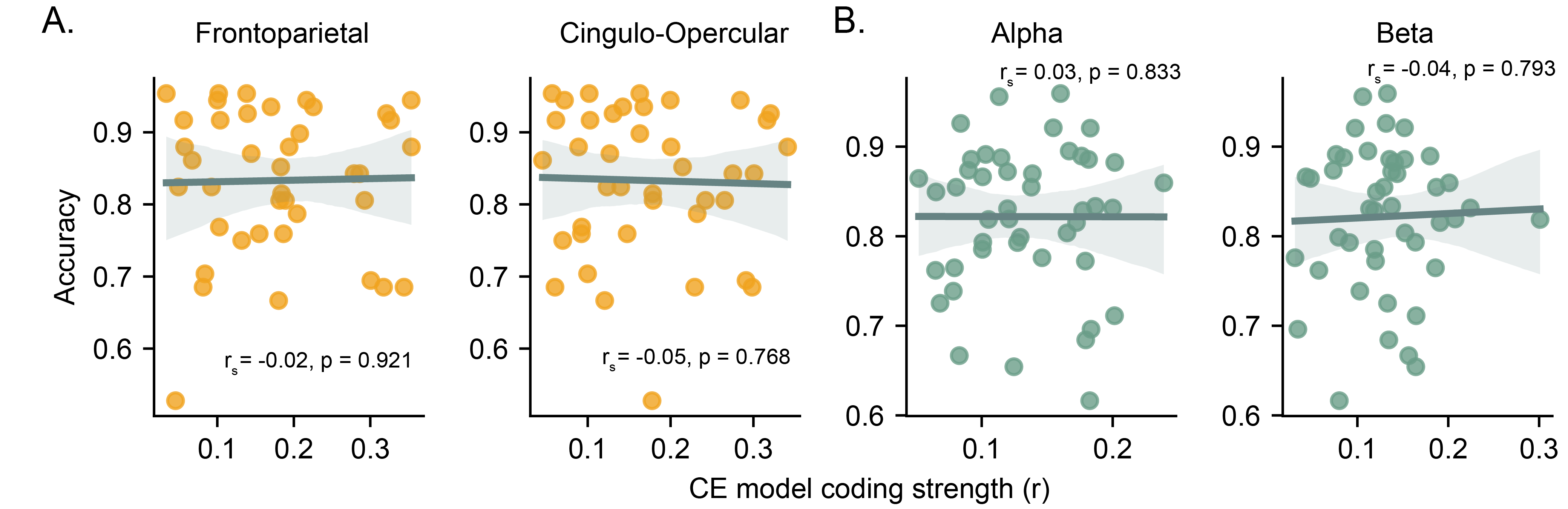


**Supplementary Figure 3**: *Correlation between participant accuracy (y-axis) and CE model coding strength (x-axis)*. **A.** fMRI CE model coding strengths for the frontoparietal (left) and cingulo-opercular (right) networks. **B.** EEG frequency-derived peak CE model coding strengths in the alpha (8 – 12 Hz, left) and beta (13 – 30 Hz, right) bands. rs represents Spearman's rank correlation coefficient; p-values are uncorrected.


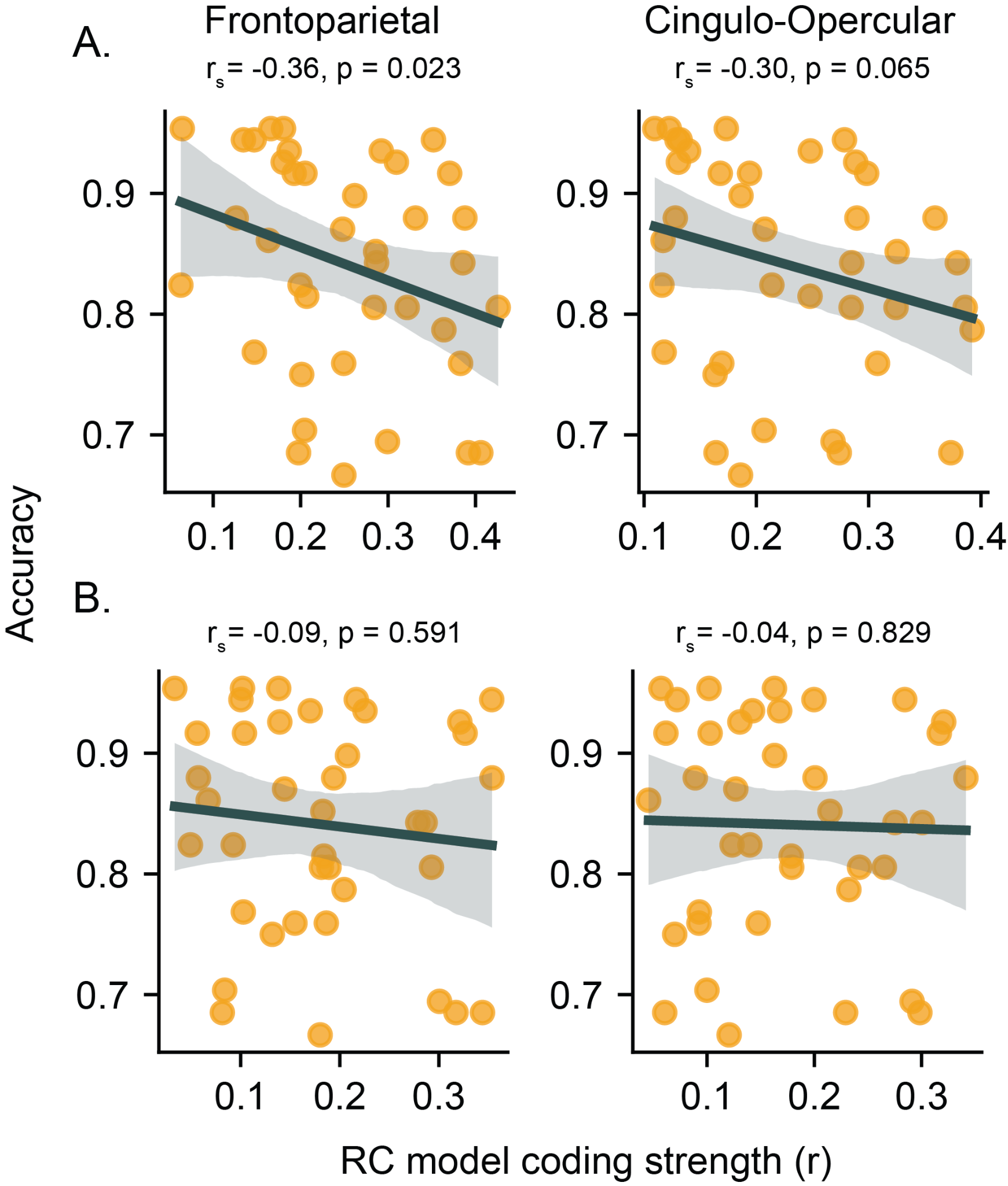


**Supplementary Figure 4.** *Correlation between participant accuracy (y-axis) and fMRI RC model coding strength (x-axis) with corresponding brain activation across complexity levels (n = 39).* For this control analysis we removed one outlier (defined as accuracy < 0.55, representing three standard deviations from the pooled mean across both fMRI and EEG datasets). **A.** RC model, **B.** CE model.

**Supplementary Table 1:** *Demographic and task-related characteristics of included and excluded fMRI participants*

|  | Included (n = 40) | Excluded (n = 22) | p-value |
| --- | --- | --- | --- |
| Age^a^ | 23.2 (3.6) | 22.9 (3.3) | 0.736 |
| Sex (m/f)^b^ | 16/24 (60%) | 13/9 (41%) | 0.149 |
| Years of education^a^ | 16.2 (1.9) | 15.6 (2.0) | 0.261 |
| *Sudoku experience^c^ | 2 (1 - 3) | 2 (2 - 2) | 0.703 |
| ^Motivation^c^ | 4 (4 - 5) | 4 (4 - 5) | 0.127 |

^a^Mean (Std) - independent t-test, ^b^Count (% female) - Chi-Squared test, ^c^Median IQR(25^th^ - 75^th^) Mann-Whitney

*Participant rating of sudoku experience from 1 to 5, 1 being low (never played sudoku), to 5 (daily sudoku use).

^Participant motivation rating to complete the task, where 1 = not motivated at all, 5 = very motivated.
